# Supplementary material for: Characterization of a member of the CEACAM protein family as a novel marker of proton pump-rich ionocytes on the zebrafish epidermis
Source: PLoS One. 2021 Jul 12;16(7):e0254533. doi: 10.1371/journal.pone.0254533 (PMC8274849; doi:10.1371/journal.pone.0254533)
Supplement: S1 Fig — (PDF) [file pone.0254533.s001.pdf]

SUPPORTING INFORMATION

Characterization of a member of the CEACAM protein family as a novel marker of proton pump-rich ionocytes on the zebrafish epidermis

Julien Kowalewski[1,#], Théo Paris[1,#], Catherine Gonzalez[1], Etienne Lelièvre[1], Lina Castaño Valencia [1], Morgan Boutrois[1], Camille Augier[1], Georges Lutfalla[1,*], Laure Yatime[1,*]

[#] These authors contributed equally to the work

Affiliation

[1] LPHI UMR5235, Univ. Montpellier, CNRS, INSERM, F-34095 Montpellier, France

***Correspondence to**

Dr. Laure Yatime (email: laure.yatime@inserm.fr) or Dr. Georges Lutfalla (email: georges.lutfalla@umontpellier.fr)

CEAz1fl_Fw1 →

5' AATATTAGCAGTATGAAAGTATAGCGATGATTAGTTTCGGACACGTCTGAGGTCTGAGGAAGAAGTCAGGGATACTCTAGTTAGGATTTCCAAA -1

ATG GGA TAT AAA GTT CTT GCC TTC TTC GTC TTA TTC TGT GCA CCA GGT CTT TGT CAA GTG AAT GTG GTT CCA 72
Exon 1

TCA AAT AAC CCA GTG GCA GTC GGG AGC AAT GTC ACC CTA GAT GTG AAT TCG TCC ATG CCC ATT ACA GTT GGG 144

CTC TGG TTG TTT GGG CCT AGT ACA TTG TTC ATG TGG TAC ATG GGG GAC ATT ATT CCG GGT AAT AGC CTC CAA 216

CCG GGA GTA TAT TTT AAC AGC TCT ACA TAT CAG CTC ACC TTA TCG GCA GTA ACC CTG GAG AGT TCT GGC GTG 288

TAT GTG CTG GAT GTA TAT AAA CCC AAC CGG ATC AGA TCA GAG ATT ACG TTG GAA GTT CAG GAA CCC GTC GGT 360
Exon 2

AAC GTA AAT ACA ACT GTA AAC ACA ACA AAC CTG GTG GAG GTG AAC GAC ACC GTG TAC TTC ACC TGC TCG GTT 432

ACA ACT TCG CCG GTG TGG TTT TCG TGG CTG AAC GGC AGC TCT GCG GTC AAA GAT GGA GGG AGA GTT CAG CTC 504

CEAz1seq_int1 →

CAA AAC AGT GGA CAA ACT CTT GTA ATC AAC GGA GTG ACT CGG TAC GAT GAA GGA CCA TTC AAG TGT GTT GTG 576

GTA AAC AAC ATC AGC AGC CAG CAG AGT GTT GAC ATG AAA CTC AAT ATA AGC TAT GGG CCC GGA AAC CTG ACA 648
Exon 3

CTT ACT GCC ATG CCA GAG AAA ACA GTA TAC ATT TCT GGT TCA AAC TTT TCT CTG TCA TGC TCT GCT GAC TCC 720

AAA CCG ACA GCT ACT TTT AAC TGG ATG TTG AAT GGC AAT CTT CTG AAT GAC AAT GGT CCG GTT TAC GTA TTT 792

ACA AAG GCT ACT CAA AAC CAG TCG GGA GTG TAC ACC TGT GGT GCC CAA AAC GCA GCA ACA CTC AGA TAT GCT 864

GCA GTG ACA AAA ACT ATT CGT ATA GTT GAT CCA ATA TCA GAA GTG GTC GTG AAT TCA ACA AGC TTC CCA GTT 936
Exon 4

GAA AAT GTA CCT TTT AAT CTA AAA TGT AAT GTT GTG GGA CCA GTG GAC TCC ATT CAG TGG ATG AAG GAT GGA 1008

GTG TAC CTG TAT ACT GAC AAC ACA ACC ACG CTC TCT AGT GAC AAC TCA ACC CTG AGC TTT AAA CAA CTC GCT 1080

CTT AGT GAT GAC GGA CTG TAC CAG TGC ACA GCT AGT AAT GCA GTG AGC GAC ATG ACC CAG GCC TAT AAC CTG 1152

CEAz1-ISH_Fw1 →

ACT GTC AAC TAT GGC CCA ATC AAC ACA GCG GTC TCC GGT CCA TTT GTA GCA GCA GTG GGA GAT AGT GTG ACC 1224
Exon 5

CEAz1seq_int2 →

TTC AGC TGT TCC TCT AAC TCT CGT CCT CAA AGT CAG TAC AGC TGG TAT TTC AAT GGC TTT AAT GTG TTC AAC 1296

GGC CCA GTG TAC GTA ACT GCA GCT CTC CTA CAA AAC CAA AGT GGA CTG TAC ACC TGC ATG GCC TTT AAT AGT 1368

ATC ACA GGC AAA ACC AAC AAC AAT TCA ATG ACA TTA ACC GTC CTT GTT CCT GTG AGC AAT GTT GTG GTG AAT 1440
Exon 6

ATT AGC GAT GGA CAG CAA CCA ATC TTC AGT AAC CCA TTC ACA TTA ACC TGC ACG GCC AGT GGA AAT GTT GAT 1512

TAC ATT CAG TGG ATG TTG GAC GGC GCA GTC CTT TAT CCT CAA GAT GGA ATC ACT TTC TCT GGT GAC AAC TCA 1584

ACC TTG AGC TTC AGT AAT CTC ACT CTC AGG GAT AAT GGA AAT TAT CAG TGT GAA GCA AGT AAC GAT ATC AGC 1656

AAT ATG ACC AGC ACA GCC TTT GAC TTG ATG GTC AAC TAT GGT CCA TGG AAC GTA ACA CTT AAT GGC CCA AAC 1728
Exon 7

ATG GCA AGA GCT GGA TCC ACT GTG ACT TTC AGC TGC ACT GCG GAT TCT TAT CCT GCA AGT CAG TTC AGC TGG 1800

TTC TTT AAC AGC TCG TGG GTG GGA AAT GGT CCA GTG TAT GTG ACT GCA CCT CTG TCA CAC AAC AGC AGT GGA 1872

CAG TAT ACA TGC ATG GCC TTC AAT GCC ATA ACA GGC AGC AAC AGC AGC TCT TCA GCT GTG CAG TTA AAT GTA 1944

CEAz1seq_int3 →

ATT GAT CCA GTT AGT AAT GTT GTT GTG AAC GTG GGC AAT CAG CAA CCA GTC TAT AAT CAA CCA TTC ACA CTA 2016
Exon 8

ACC TGC ACT GCC AGT GGA AAT GTT GAT TAC ATT CAG TGG GTG TTG AAC GGC ACA GAC CTT CTT CCT CAC GAT 2088

GGA ATC ACT TTC ACT AGT GAT AAC TCA ACC TTG AGC TTC ATC AAT CTC ACA CTC AGT GAT AAC GGA CAT TAT 2160

CAG TGT GAA GCA AGT AAC GAG ATC AGC AAT ATG ACC AGT TCT GCA TAT GAC CTG GTG GTC AAC TAT GGT CCA 2232
Exon 9

TGG AGG GTA ACA ATT AAT GGC CCA AGC ATG GCA GAA ACA GGA TCC AGT GTG ACT TTC AAC TGC ACT GCT GAT 2304

TCT CTT CCT GCA AGT CAG TTC AGC TGG TTC TTC AAC AGC TCA TGG GTG GGA AAT GGT CCA GTG TAT GTG ACT 2376

GCA CCT CTG TCA CAA AAC AGT ACT GGA CAG TAC ACT TGC ATG GCC TTC AAT GCC ATT ACA GGC ATC AAC AGC 2448

AGC TCT TCA GCT GTG CAG TTA ACT GTA ATT GAT GCC ATC ACC ACA GTG GAC GTG ACG GCC AGT CCT CTC ATT 2520
Exon 10

CCT CTG GTT TCT AAA AGC CTG CAG CTC ACC TGT AAT GTG AAT GGA CCC TAC ATC AAT CTT CAC TGG CTT CGA 2592

AAC AAC AAT AAT TTC TTC CCA TCA AAC AGA ATT ACG TTT TCT GCT GAC AAC ACC ACA GTG ACC TTC AGT TCA 2664

TTG CAA ACC ACT GAT GAT GGA AGT TAC CAG TGT GTC GCC GCC AAT GCA ATA AGA CAT CAC ATC AGT AAT CCA 2736

TAT AAA CTT ATG GTC ATC TAT GGA CCA CAA AGC GTG CAG ATC ACT TTG CAT CCT GGG ATT CCT CCA GTC CTG 2808
Exon 11

ACA TGT CTA GCA GTG TCT CAG CCA CCC TCT GTG TAT TAC TGG ATA CTC GAC AAC AAC ACA GTT GTG GGG GAT 2880

CAA GCT TCC ATT ACG ATT CCT ATT ACA TCT ATC CTG GGC AGC AAT TAC ACC TGT GTG GCC AAA AAC CCT CTG 2952

← CEAz1-ISH_Rv1

ACC AAC TTG ACT CTC TCC ATC AGC CAA GTC GTC AGC TAT CCC AAC GCA GCT GAC AGA TTC CAG GCC AGT CAT 3024
Exon 12

ATG CTG ATG GGC CTC CTC GTT CTG CTG TTC TCT TTA CTA CAG GAA TGG CTG TAA TTCTGAGACTGCTCTCAGGC 3' 3075

← CEAz1fl_Rv1 Exon 13

S1 Fig. Complete DNA sequence of the *ceacamz1* coding region. The same color code as in main Figures 1 and 2 is used to delineate the different CEACAMz1 domains. The ATG start codon and the TAA stop codon are highlighted in red and dark blue, respectively. The ending point for each of the different exons of *ceacamz1* is indicated below the sequence. All exons are symmetrical and are separated by phase 1 introns. Primers used to amplify the full-length *ceacamz1* coding region are highlighted in light orange. They were also used for sequencing purpose as well as the primers highlighted in pink. Primers used to construct the RNA probes for ISH are shown in green.
