# Supplementary material for: Characterization of a member of the CEACAM protein family as a novel marker of proton pump-rich ionocytes on the zebrafish epidermis
Source: PLoS One. 2021 Jul 12;16(7):e0254533. doi: 10.1371/journal.pone.0254533 (PMC8274849; doi:10.1371/journal.pone.0254533)
Supplement: S2 Fig — (PDF) [file pone.0254533.s002.pdf]

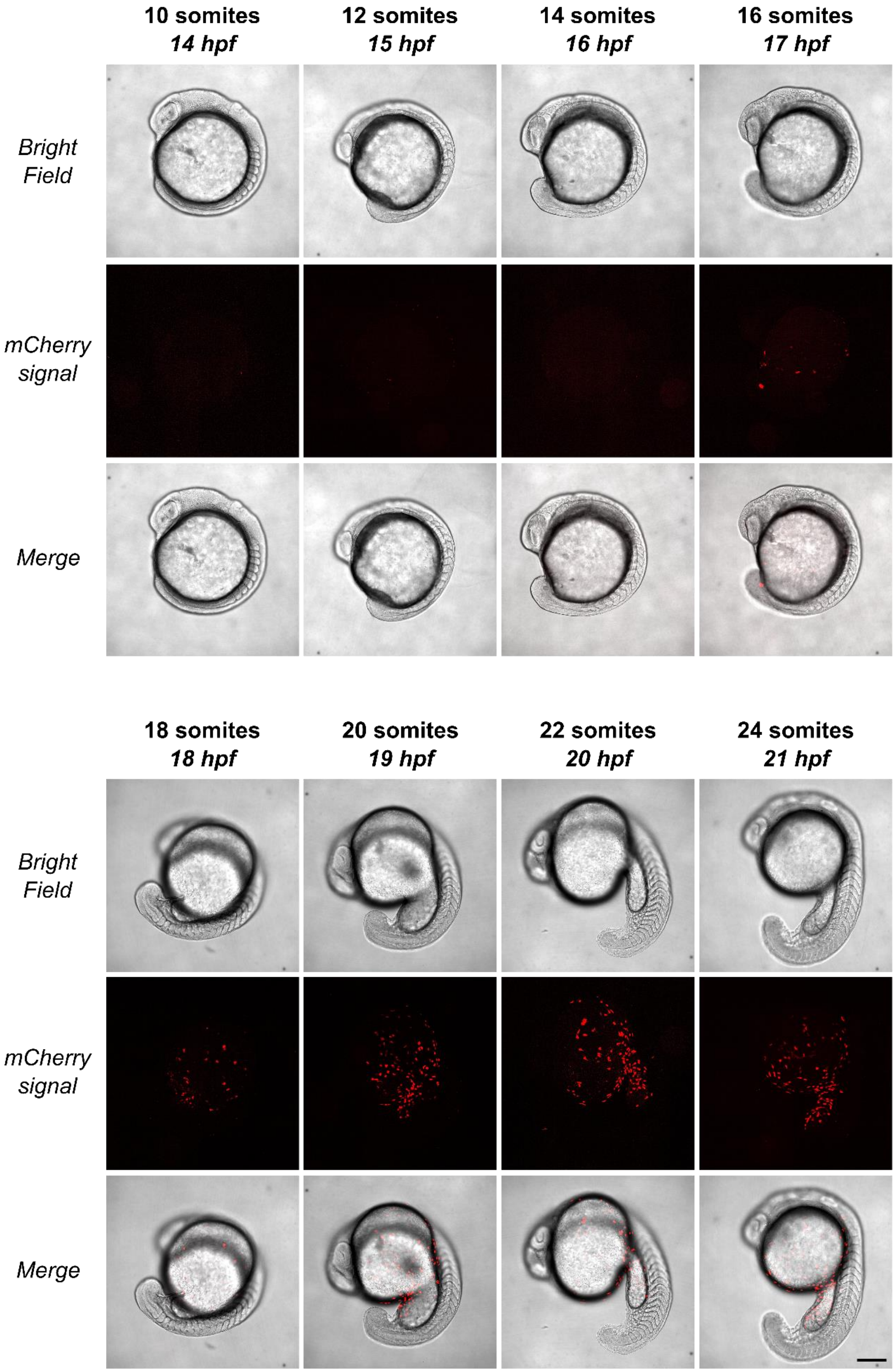
10 somites
14 hpf
12 somites
15 hpf
14 somites
16 hpf
16 somites
17 hpf
Bright Field
mCherry signal
Merge
18 somites
18 hpf
20 somites
19 hpf
22 somites
20 hpf
24 somites
21 hpf
Bright Field
mCherry signal
Merge

S2 Fig. Imaging of the transgenic line *Tg(ceacamz1:mCherry-F) ump9Tg* from 10 to 24 somites stages. Embryos from the ceacamz1 trangenic reporter line *Tg(ceacamz1:mCherry-F)* were imaged using spinning disk confocal microscopy (10x/0.25 Air objective) after immobilization in agarose at different developmental stages between 10 and 24 somite stages (14 to 21 hpf). For the mCherry signal, Z-stacks were recorded over the embryos. Corresponding maximum intensity Z-projections are displayed in the central panel for each developmental stage. Bright field images are shown on the upper panel and merged images (bright field + mCherry signal) are shown in the lower panel. To insure that embryos that did not show fluorescence signal at early developmental stages were still positive for fluorescence at later stages, these embryos were recovered from agarose and incubated again at 28°C until fluorescence signal could be detected. These embryos were however not used again for imaging as the recovering procedure may have slightly damaged them. mCherry fluorescent signal could clearly be detected from 16 somites stage and later on, on the yolk region and yolk extension. The scale bar indicates 0.2 mm.
