# Supplementary material for: Characterization of a member of the CEACAM protein family as a novel marker of proton pump-rich ionocytes on the zebrafish epidermis
Source: PLoS One. 2021 Jul 12;16(7):e0254533. doi: 10.1371/journal.pone.0254533 (PMC8274849; doi:10.1371/journal.pone.0254533)
Supplement: S1 Raw images — (PDF) [file pone.0254533.s003.pdf]

1 kb DNA ladder, New England Biolabs (N3232S)

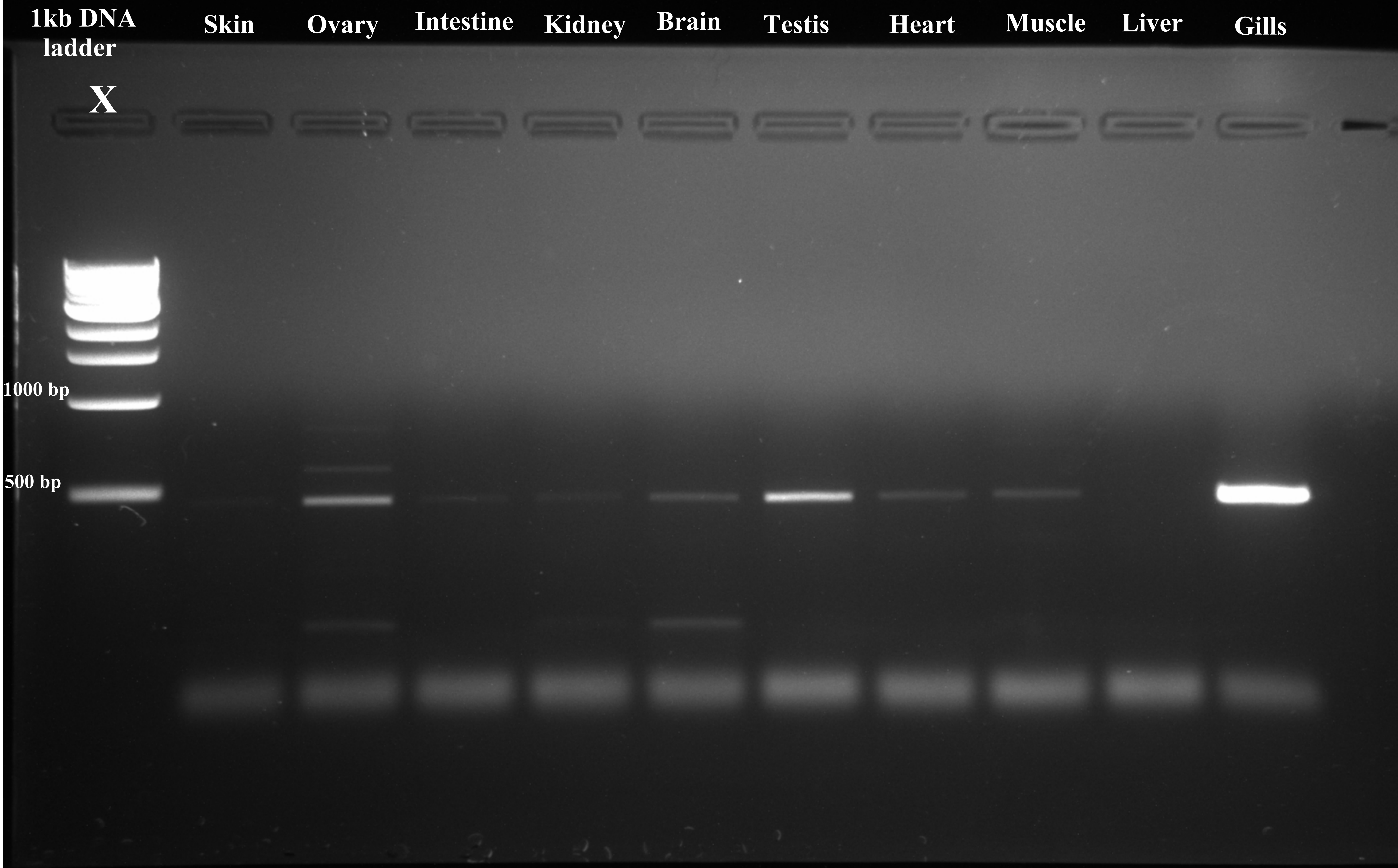

Gel captured with an Ingenius 3 imaging system (SYNGENE) using GeneSys image capture software

This gel was used to generate Figure 3 panel A, upper image: CEACAMz1 RT-PCR in various adult tissues

1 kb DNA ladder, New England Biolabs (N3232S)

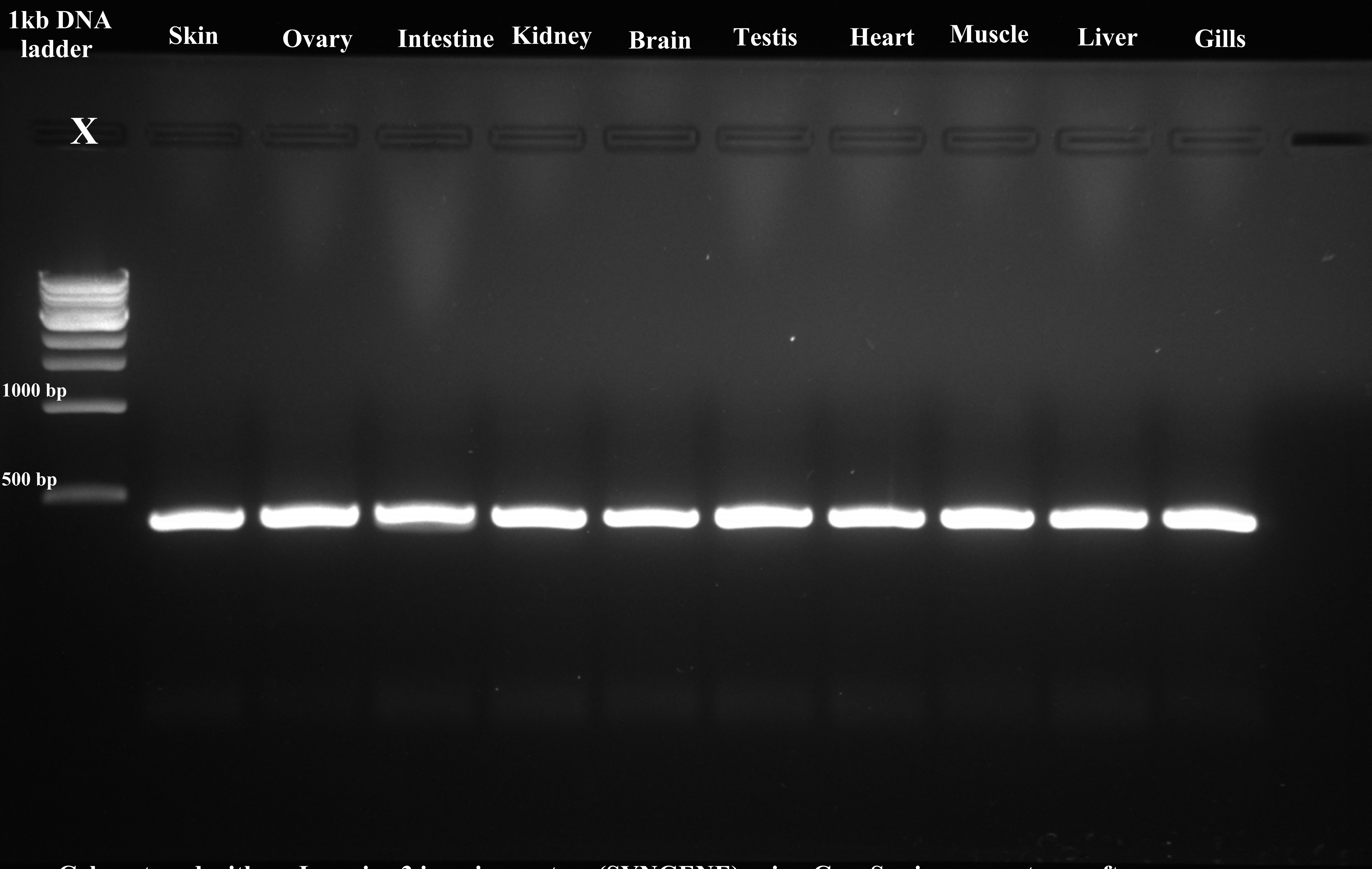

Gel captured with an Ingenius 3 imaging system (SYNGENE) using GeneSys image capture software

This gel was used to generate Figure 3 panel A, lower image: RACK1 RT-PCR in various adult tissues

100bp DNA ladder, New England Biolabs (N3231S)

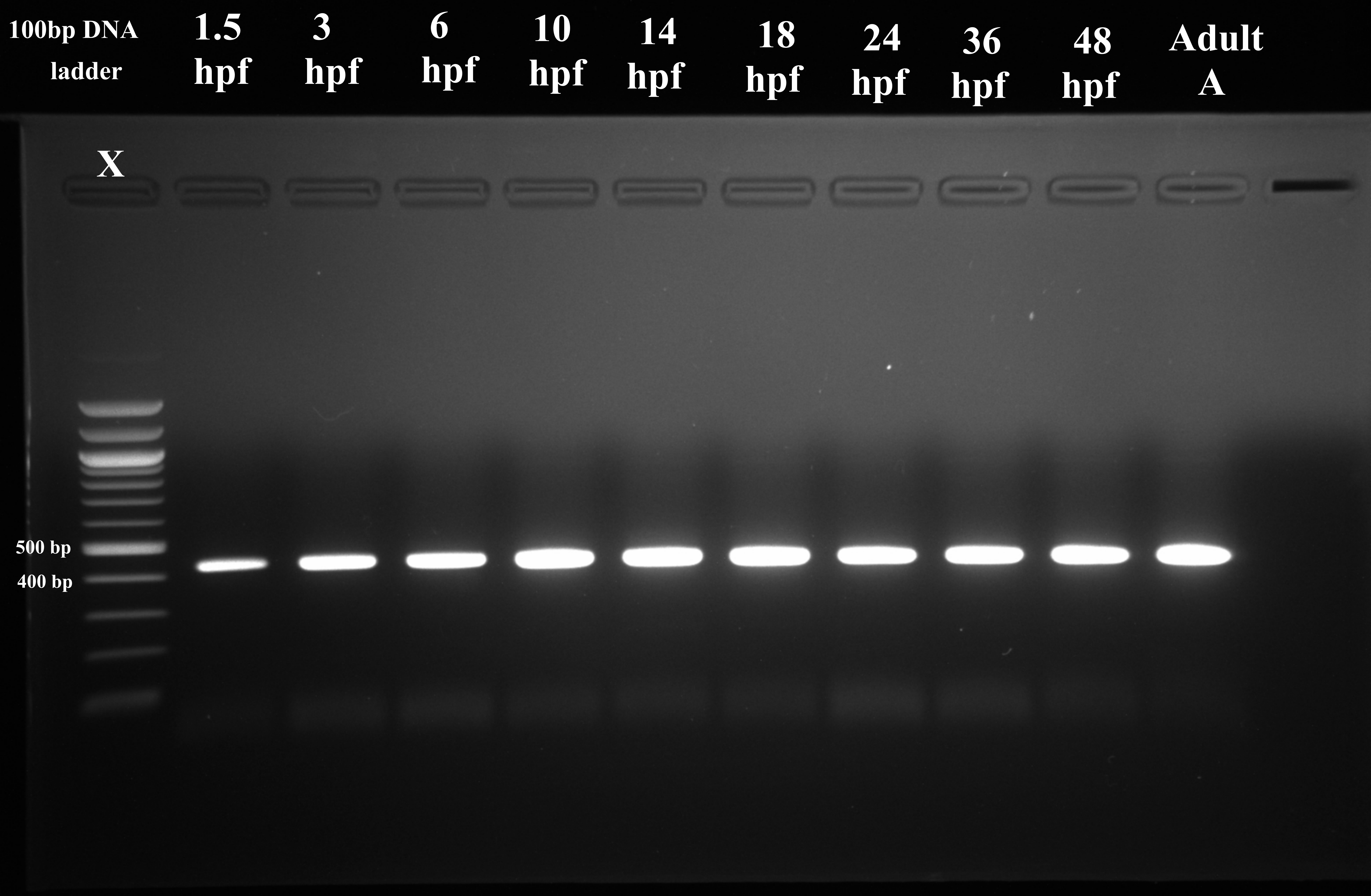

Gel captured with an Ingenius 3 imaging system (SYNGENE) using GeneSys image capture software

This gel was used to generate Figure 3 panel B, lower image: RACK1 RT-PCR at various developmental stages

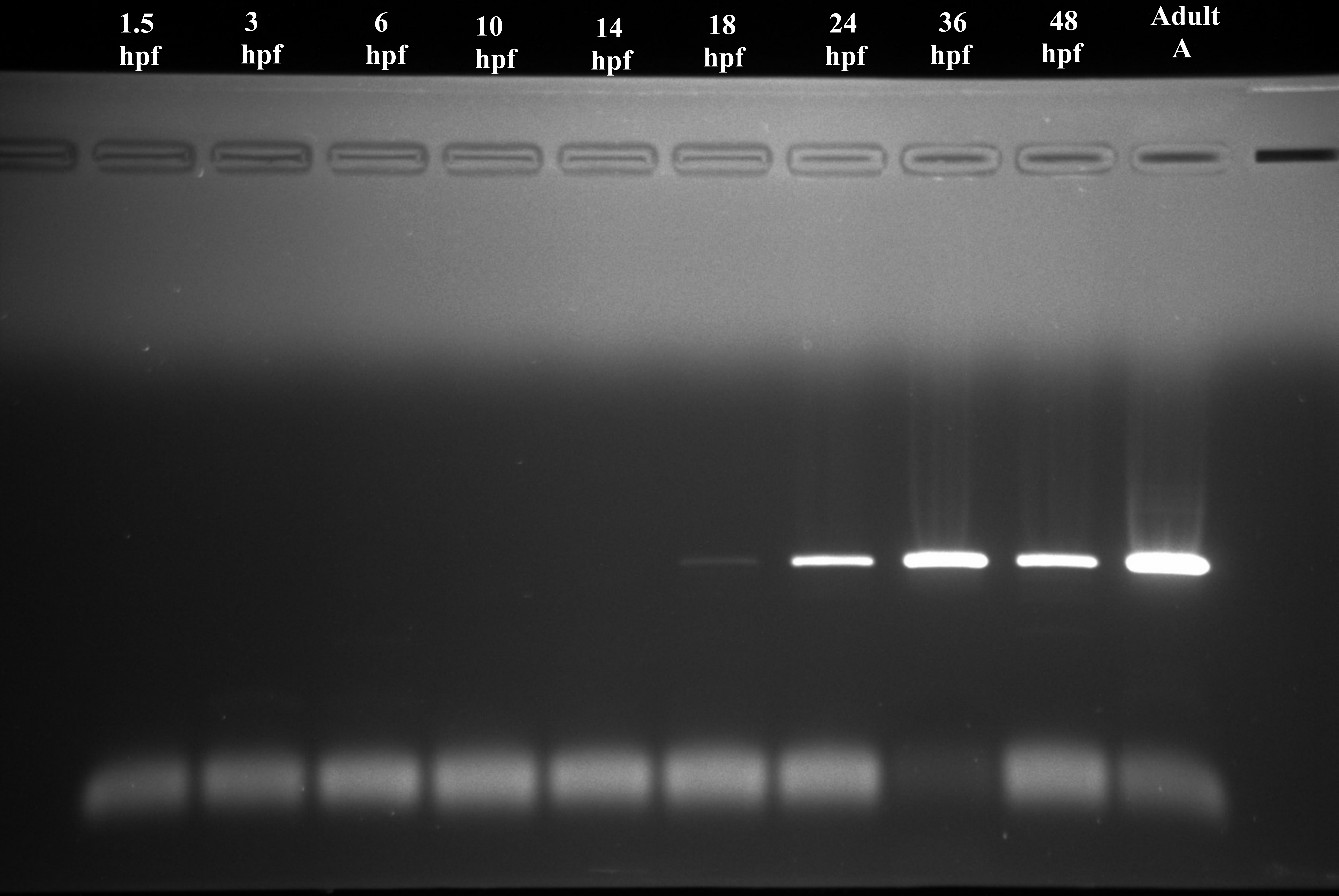

Gel captured with an Ingenius 3 imaging system (SYNGENE) using GeneSys image capture software

This gel was used to generate Figure 3 panel B, upper image: CEACAMz1 RT-PCR at various developmental stages
